# Estrogen: the forgotten player in metaflammation

**DOI:** 10.3389/fphar.2024.1478819

**Published:** 2024-11-07

**Authors:** Bao-Ting Zhu, Qing-Qing Liao, Hai-Ying Tian, Dao-Jiang Yu, Teng Xie, Xi-Lu Sun, Xin-Meng Zhou, Ying-Xuan Han, Yu-Jie Zhao, Mohamed El-Kassas, Xiu-Xiu Liu, Xiao-Dong Sun, Yuan-Yuan Zhang

**Affiliations:** ^1^ West China School of Pharmacy, West China School of Basic Medical Sciences and Forensic Medicine, West China School of Public Health and West China Fourth Hospital, Sichuan University, Chengdu, China; ^2^ The Second Affiliated Hospital of Chengdu Medical College, China National Nuclear Corporation 416 Hospital, Chengdu, China; ^3^ Medical College, Tibet University, Lasa, China; ^4^ Endemic Medicine Department, Faculty of Medicine, Helwan University, Cairo, Egypt; ^5^ Liver Disease Research Center, College of Medicine, King Saud University, Riyadh, Saudi Arabia; ^6^ Steatotic Liver Disease Study Foundation in Middle East and North Africa (SLMENA), Cairo, Egypt

**Keywords:** estrogen, metaflammation, metabolic inflammatory syndrome, network pharmacology, pharmacological targets, sex differences, antiinflammatory effects, hormone replacement therapy (HRT)

## Abstract

Metaflammation is low-grade inflammation triggered by chronic metabolic imbalance and caused by dysregulated metabolites in metabolic inflammatory syndrome (MIS), which includes four diseases: obesity, type 2 diabetes mellitus (T2DM), atherosclerosis (AS), and nonalcoholic fatty liver diseases (NAFLD, recently proposed to be replaced by metabolic dysfunction-associated steatotic liver disease, MASLD). These diseases exhibit apparent sex dimorphism as regards MIS. Estrogen not only plays a crucial role in gender differences in adults but also possesses an anti-inflammatory effect on many metabolic diseases. In this study, we present a prediction of the differential proteins and signal transduction of estrogen in MIS through network pharmacology and review the validated studies on obesity, T2DM, AS, and NAFLD. Subsequently, we compared them to obtain valuable targets, identify current gaps, and provide perspectives for future research on the mechanisms of estrogen in metaflammation.

## Graphical Abstract

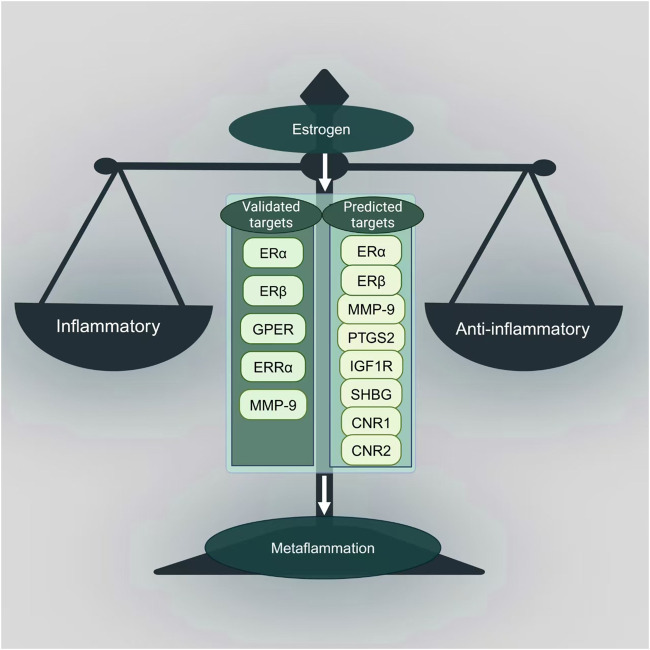

## 1 Introduction

Habitual overnutrition caused by the modern diet and the sedentary lifestyle results in excess energy intake and decreased energy expenditure, leading to chronic metabolic inflammation, termed metaflammation (Christ et al., 2019). Metaflammation was first proposed in 2006 by Hotamisligil, who deemed that although inflammation can promote the deterioration of metabolism, in the case of excessive metabolism, metabolic signals are more likely to be the trigger of the inflammatory response, and then further impair the metabolic function, resulting in more inflammation (Hotamisligil, 2006). Some proposed mechanisms are linking metabolic diseases and inflammation. From an evolutionary point of view, ancestors tried their best to seek out and ingest food to meet their energy needs and prevent life-threatening hypoglycemia. However, with the diminishing selective pressure, they shifted from metabolic adaptations to obesity and related metabolic diseases such as diabetes (Hotamisligil, 2017). Accordingly, metaflammation could be defined as a metabolic imbalance-induced immune abnormalities. The initiating factors of traditional inflammatory responses are pathogens such as microorganisms, parasites, or molecules of injured tissues, with redness, swelling, heat, and pain as the main gross symptoms (Gregor and Hotamisligil, 2011). In metaflammation, excess nutrients, including lipid metabolites such as oxidized low-density lipoprotein, high amounts of glucose, glycosylated end products, etc., work as inflammatory substances and induce trained immunity (Christ et al., 2019).

Metaflammation is the root of metabolic disorders, including obesity, type 2 diabetes mellitus (T2DM), atherosclerosis (AS), and nonalcoholic fatty liver disease (NAFLD, recently proposed to be renamed metabolic dysfunction-associated steatotic liver disease, MASLD) (Renming et al., 2022; Rinella et al., 2023). These four diseases, closely associated with chronic low-grade inflammation, are often aggregated, co-existed, or concurrent. Anti-inflammatory treatment yielded beneficial metabolic effects in the four diseases, suggesting they can be prevented and treated simultaneously (Renming et al., 2022; Hu R. et al., 2016). Hence, based on clinical and basic studies, Hu et al. proposed a novel concept, metabolic inflammatory syndrome (MIS), and suggested that patients with two or more of the above four metabolic diseases are to be diagnosed as MIS (Hu R. et al., 2016).

The incidences of obesity, T2DM, AS, and NAFLD exhibit noticeable sex differences (Regensteiner and Reusch, 2022; Yerly et al., 2023; Cherubini et al., 2024). Premenopausal women are generally more protected from these diseases than men or postmenopausal women (Man et al., 2020; Balakrishnan et al., 2021; Sun et al., 2023). According to the United Nations World Population 2021 standardized global diabetes prevalence statistics for adults aged 20–79 years, although the difference in prevalence between men and women aged 20–79 years was not significant (10.8% for men vs*.* 10.2% for women), the prevalence of diabetes was higher in men than in women aged 25–69 years (Sun et al., 2023). Similarly, non-invasive imaging and pathological evaluation showed that AS plaques appear earlier in men than women. Moreover, plaques in males are more inflamed and show more unstable characteristics than females (Man et al., 2020). A meta-analysis including 62,239 subjects reported that the risk of NAFLD in women was 19% lower than in men (Balakrishnan et al., 2021). However, these “female advantages” usually disappear after menopause. Since sex hormones are the major contributor to gender differences in adults, we speculate that estrogen, the primary sex hormone possessing metabolism regulation and anti-inflammation effects, plays an essential role in MIS.

Estrogen exists in various forms including estrone (E1), 17β-estradiol (E2), estriol (E3), and estetrol (E4) (Stanczyk and Archer, 2023). E2 is the main estrogen in women during the childbearing period and is mostly produced by the ovary (Stanczyk, 2024). However, E1 becomes the main estrogen in postmenopausal women (Xu et al., 2022), while E3 and E4 are mainly produced by the placenta during pregnancy (Stanczyk and Archer, 2023). In males, testosterone is metabolized to estrogen by aromatase in the testicle and other tissues (Naamneh Elzenaty et al., 2022). Estrogen, which exerts the effects through the classical estrogen receptors alpha (ERα), ER beta (ERβ), and the G protein-coupled estrogen receptor (GPER, also known as GPR30), not only plays a pivotal role in regulating a multitude of physiological processes, including female sexual development, the menstrual cycle, and reproduction, but also exerts control over the metabolism of glucose, lipids, and bone (Patel et al., 2018; Arterburn and Prossnitz, 2023). Estrogen is supposed to play a key role in metaflammation and MIS, possibly by regulating metabolism and inhibiting inflammation to prevent the development of insulin resistance (IR)(De Paoli et al., 2021). Hormone replacement therapy (HRT) is widely used in menopause syndrome and related diseases. HRT, mainly estrogen, was shown to mitigate MIS by reducing the risk of cardiovascular disease, diabetes, and insulin resistance and improving adverse alterations in the lipid-lipoprotein profile (Goldštajn et al., 2023). However, HRT is a double-edged sword and has side effects, such as increasing the risk of stroke, venous thrombosis, and even breast cancer (Voedisch, 2023). It is, therefore, of overwhelming importance to delve into the mechanisms of estrogen in MIS and to determine its pharmacological targets, which will provide novel strategies and pharmacological targets for metaflammation in MIS. In this study, we are predicting and identifying the targets and pathways of estrogen’s role in metaflammation in MIS by an *in silico* study and reviewing the currently available evidence for validation. As for the *in silico* study, we predicted the targets of estrogen on inflammation in obesity, T2DM, AS, and NAFLD through the network pharmacology databases, constructed a protein-protein interaction (PPI) network (Martino et al., 2021), and carried out Gene Ontology (GO) enrichment analysis (Consortium, 2019) and Kyoto Encyclopedia of Genes and Genomes (KEGG) pathway analysis (Kanehisa et al., 2023). As for validation, we reviewed the original studies on estrogen in the four diseases included in MIS. Finally, we compared the network pharmacology prediction results and the available experimental evidence, summarized the gaps, and proposed perspectives.

## 2 In silico study of estrogen for metaflammation in MIS

### 2.1 PPI network construction, GO enrichment, and KEGG analysis

We established a PPI network with estrogen-regulated differentially expressed proteins in MIS. Firstly we predicted the gene targets of estrogen through PubChem (https://pubchem.ncbi.nlm.nih.gov/) (Kim et al., 2022) and SwissTargetPrediction (http://swisstargetprediction.ch/) (Daina et al., 2019) databases and obtained disease gene targets through GeneCards (https://www.genecards.org/) (Stelzer et al., 2016) and the online Mendelian inheritance in man (OMIM, https://omim.org/) (Amberger et al., 2019) databases. Subsequently, Jvenn (https://jvenn.toulouse.inra.fr/app/example.html) (Bardou et al., 2014) was used to obtain the intersection target. STRING12.0 (https://cn.string-db.org/) (Szklarczyk et al., 2023) and Cytoscape3.9.1 (http://www.cytoscape.org) were used to construct the PPI network diagram. Finally, GO enrichment analysis and KEGG pathway analysis were performed through DAVID 2021 database (https://david.ncifcrf.gov/) (Sherman et al., 2022) and the bioinformatics mapping tool named SRplot (https://www.bioinformatics.com.cn/srplot) were used (Tang et al., 2023).

#### 2.1.1 Acquisition of estrogen’s targets related to MIS

To obtain accurate information on estrogen targets, we input the terms of estrogen, including estradiol, estrone, and estriol, to PubChem to obtain relevant simplified molecular input line entry system (SMILES) numbers. Next, the potential targets of estrogen whose probability is over 0 were predicted using the SwissTargetPrediction database by inputting SMILES numbers. Finally, the predicted targets obtained by estradiol, estrone, and estriol were summarized and deduplicated, from which the final estrogen-target data set was obtained.

#### 2.1.2 Acquisition of predicted targets on metaflammation in MIS

The disease targets of MIS were collected by searching the OMIM and the GeneCards database. The database search used the term “NAFLD” rather than “MASLD” to ensure comprehensive coverage of the search results. The keywords “obesity,” “type 2 diabetes mellitus,” “atherosclerosis,” “nonalcoholic fatty liver disease,” and “inflammation” were put into OMIM and GeneCards databases with the retrieval species set as “*Homo sapiens*.” After the disease targets were successfully obtained in these databases, these targets were merged, duplicate targets were removed, and the targets of the four diseases were intersected with the targets of inflammation through the Jvenn database. The results were the MIS targets we collected and used in further experiments.

#### 2.1.3 Acquisition of intersected targets of estrogen and diseases and PPI network construction

To collect the overlapping targets of estrogen and MIS, we imported the potential estrogen targets and MIS targets into the Jvenn database. The obtained intersected targets were imported into the STRING12.0 database, the interaction threshold >0.4 was set for PPI, and the free nodes were deleted to construct the PPI network diagram. Subsequently, the TSV file was obtained and put into Cytoscape 3.9.1. The three parameters of degree, closeness, and betweenness were selected as reference values through the CentiScaPe2.2 plug-in in the software, and the core targets were ultimately screened to perform network topology analysis.

#### 2.1.4 GO enrichment and KEGG pathway analysis

Based on the above data, the intersected target data sets of estrogen and the four diseases were put into the DAVID 2021 database to perform GO enrichment and KEGG pathway analysis. During the process, the official gene names were selected. The specie was limited to “*H. sapiens*,” and the analysis background was chosen as “*H. sapiens*.” The functional enrichment analysis is carried out from three aspects: biological process (BP), cellular component (CC), and molecular function (MF). After getting the relevant data, *p* < 0.05 was defined as statistical significance. The top ten results of the BP, CC, MF, and reliable target pathways with significant differences were screened out based on ranking by the number of genes. After that, the bioinformatics platform SRplot was used for visual analysis through drawing bar charts and bubble charts.

### 2.2 Results of *in silico* study

#### 2.2.1 Collection of estrogen’s potential targets and disease targets of MIS

After exporting the corresponding targets obtained on the SwissTargetPrediction database with SMILES numbers, the targets with a probability of 0 were filtered out. Then, the 87 targets obtained in total were combined and deduplicated as the final estrogen targets.

The corresponding gene information obtained in the two databases of OMIM and Genecards was combined to obtain the targets of diseases “obesity,” “type 2 diabetes mellitus,” “atherosclerosis,” “nonalcoholic fatty liver disease” and “inflammation,” and obtained 1309, 2372, 1320, 1913, and 1923 targets, respectively. After importing the respective targets of the four diseases and inflammation into the Jvenn database, 575, 880, 828, and 634 overlapping genes were obtained and considered the target genes of metaflammation in MIS.

#### 2.2.2 Intersected targets of estrogen and MIS and PPI network analysis

**FIGURE 1:**
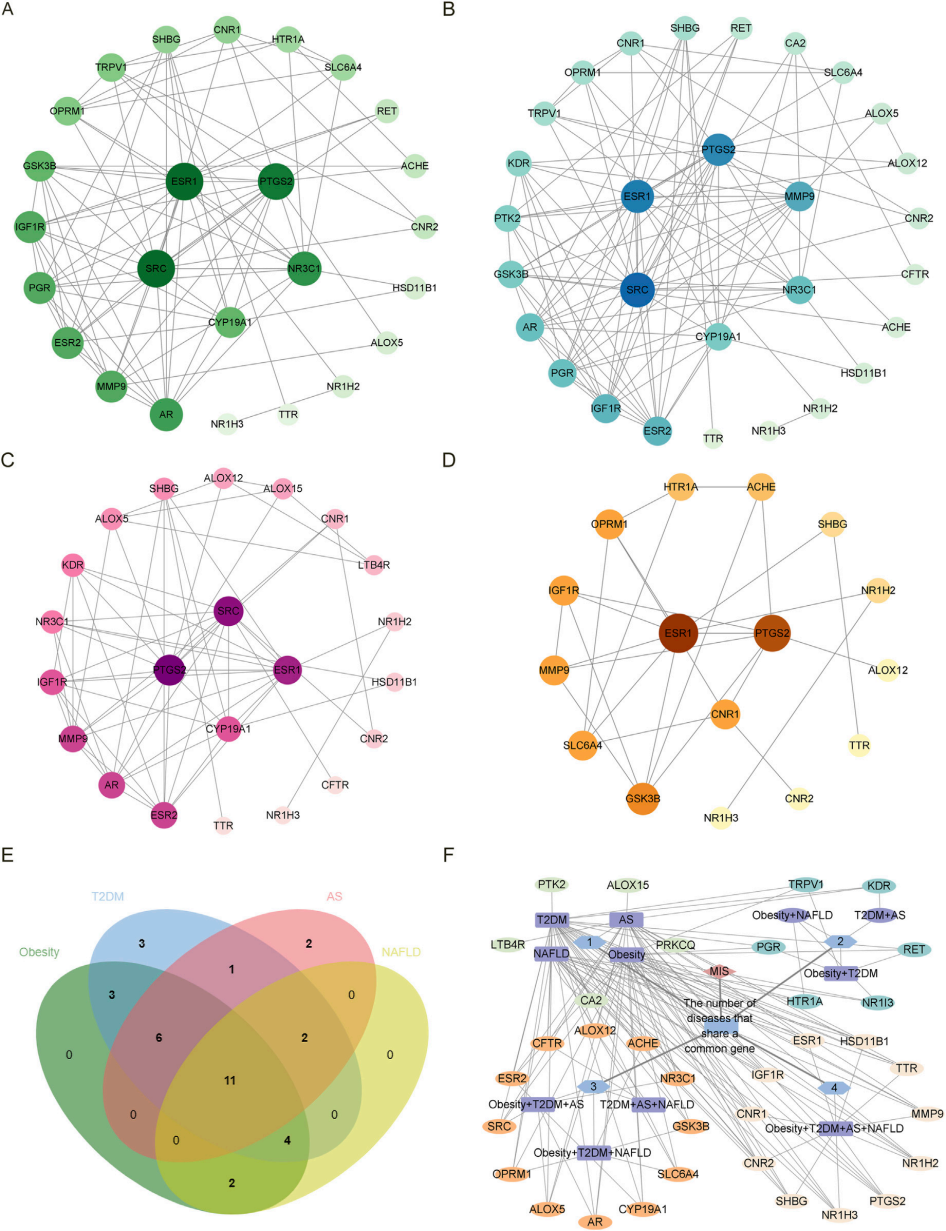
The PPI network diagram of the intersected targets of four diseases, obesity, T2DM, AS, and NAFLD, associated with estrogen. In the **(A–D)** diagram, all targets are arranged from large to small according to the degree value, and each node represents a target. The larger the degree value, the larger the node size, the darker the color, and the inner circle is the core target. NR1I3 is a free node in the PPI network of the intersection target of estrogen and obesity. PRKCQ was a free node in the PPI network of the intersection target of estrogen and T2DM. NR1I3, CFTR, and HSD11B1 are free nodes in the PPI network of estrogen and NAFLD intersection targets. The free nodes were not displayed after the PPI network was processed by Cytoscape 3.9.1. **(A)** The intersected targets of estrogen and obesity. The core targets are SRC, ESR1, PTGS2, NR3C1, and CYP19A1. **(B)** The intersected targets of estrogen and T2DM. The core targets are SRC, ESR1, PTGS2, MMP-9, NR3C1, and CYP19A1. **(C)** The intersected targets of estrogen and AS. The core targets are PTGS2, SRC, ESR1, and CYP19A1. **(D)** The intersected targets of estrogen and NAFLD. The core targets are ESR1, PTGS2, CNR1. **(E)**The common targets in the Venn diagram were obtained for the four diseases and estrogen, respectively. **(F)** The PPI network diagram of the common targets in the intersected targets was obtained for the four diseases and estrogen, respectively. The outermost layer of each combination graph is the target; the blue node shows the number of diseases with a common target, the purple node shows the disease, and the line between the disease and the target represents the relationship. Abbreviations: AS, atherosclerosis; CFTR, CF transmembrane conductance regulator; CNR1, cannabinoid receptor 1; CYP19A1, cytochrome P450 family 19 subfamily A member 1; ESR1, estrogen receptor 1; HSD11B1, hydroxysteroid 11-beta dehydrogenase 1; MMP-9, matrix metalloproteinases-9; NAFLD, nonalcoholic fatty liver disease; NR1I3, nuclear receptor subfamily 1 group I member 3; NR3C1, nuclear receptor subfamily 3 group C member 1; PPI, protein-protein interaction; PRKCQ, protein kinase C theta; PTGS2, prostaglandin-endoperoxide synthase 2; SRC, SRC proto-oncogene, non-receptor tyrosine kinase; T2DM, type 2 diabetes mellitus.

According to the Venn diagram, there are 26, 30, 22, and 19 common targets between estrogen and obesity, T2DM, AS, and NAFLD, respectively. As shown by the PPI network, there are 25 nodes and 89 edges on the targets between estrogen and obesity (Figure 1A), 29 nodes and 107 edges on the targets between estrogen and T2DM (Figure 1B), 22 nodes and 62 edges on the targets between estrogen and AS (Figure 1C), 16 nodes and 27 edges on the targets between estrogen and NAFLD (Figure 1D). The core targets between estrogen and obesity are proto-oncogene, non-receptor tyrosine kinase (SRC), estrogen receptor 1 (ESR1), prostaglandin-endoperoxide synthase 2 (PTGS2), nuclear receptor subfamily 3 group C member 1 (NR3C1) and cytochrome P450 family 19 subfamily A member 1 (CYP19A1). The core targets between estrogen and T2DM are SRC, ESR1, PTGS2, matrix metalloproteinases-9 (MMP-9), NR3C1, CYP19A1. The core targets between estrogen and AS are PTGS2, SRC, ESR1, and CYP19A1, and the core targets between estrogen and NAFLD are ESR1, PTGS2, and cannabinoid receptor 1 (CNR1). As shown in Figures 1E,F, there are 11 targets of estrogen in the four diseases of MIS through regulating inflammation, which are ESR1, sex hormone-binding globulin (SHBG), insulin-like growth factors-1 receptor (IGF1R), nuclear receptor subfamily 1 group H member 3 (NR1H3), nuclear receptor subfamily 1 group H member 2 (NR1H2), transthyretin (TTR), PTGS2, MMP-9, hydroxysteroid 11-beta dehydrogenase 1 (HSD11B1), CNR1, cannabinoid receptor 2 (CNR2). There are 12 targets of estrogen in three combinations of three diseases. The common targets of obesity, T2DM, and AS are estrogen receptor 2 (ESR2), androgen receptor (AR), CYP19A1, NR3C1, SRC, and arachidonate 5-lipoxygenase (ALOX5). The common targets of obesity, T2DM, and NAFLD are solute carrier family 6 member 4 (SLC6A4), acetylcholinesterase (ACHE), opioid receptor Mu 1 (OPRM1), and glycogen synthase kinase 3 beta (GSK3B). The common targets of T2DM, AS, and NAFLD are arachidonate 12-lipoxygenase, 12S type (ALOX12), and CF Transmembrane conductance regulator (CFTR).

#### 2.2.3 GO enrichment and KEGG pathway analysis

**FIGURE 2:**
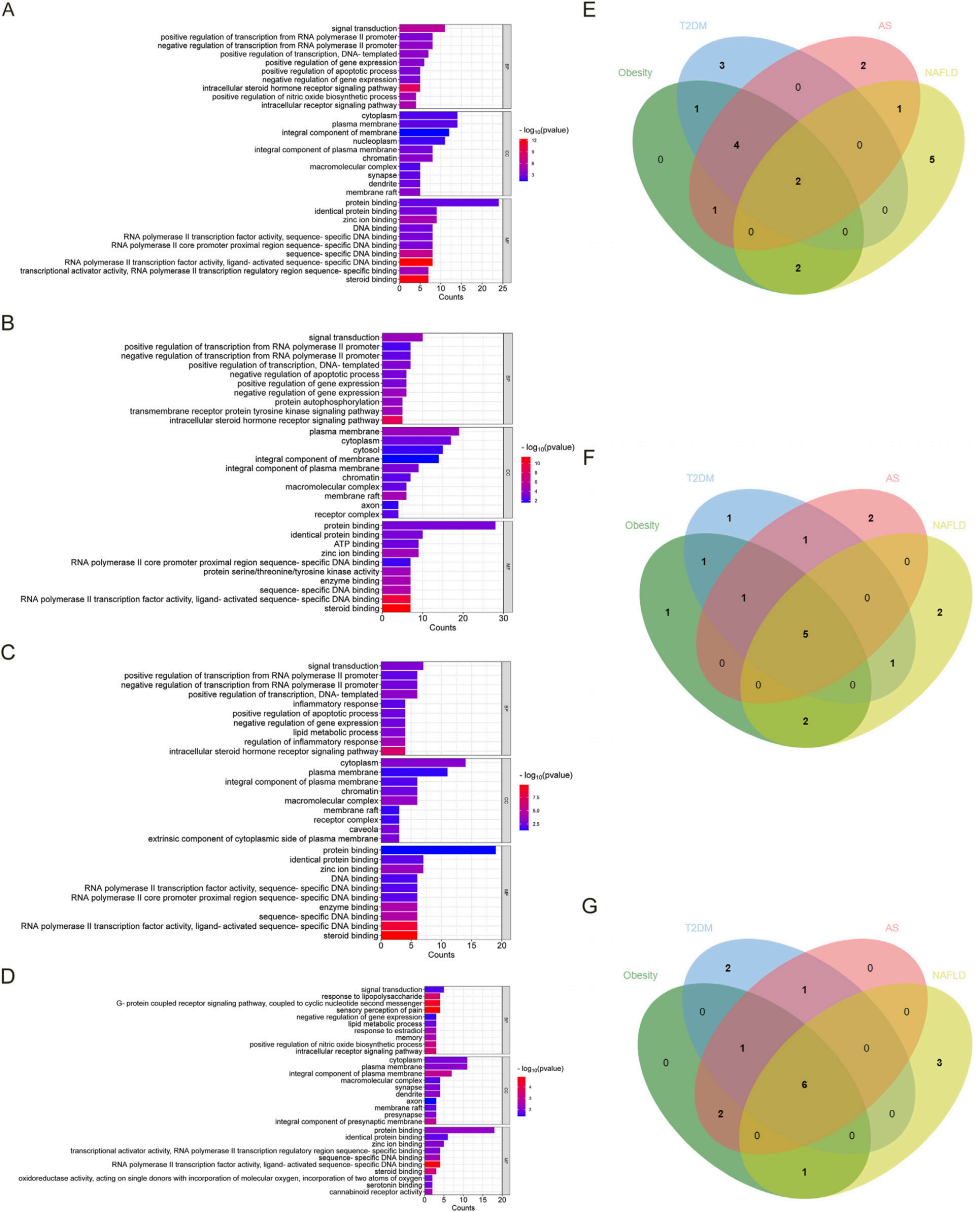
The GO analysis and Venn diagram of the intersected targets of four diseases, obesity, T2DM, AS, and NAFLD, associated with estrogen. **(A)**. The enrichment results of BP, CC, and MF of the intersected targets of estrogen and obesity. **(B)** The enrichment results of BP, CC, and MF of the intersected targets of estrogen and T2DM. **(C)** The enrichment results of BP, CC, and MF of the intersected targets of estrogen and AS. **(D)** The enrichment results of BP, CC, and MF in the intersected targets of estrogen and NAFLD. **(E)** The BP Venn diagram of the common targets contained in the intersected targets was obtained by the four diseases and estrogen, respectively. **(F)** The CC Venn diagram of the common targets contained in the intersected targets was obtained by the four diseases and estrogen, respectively. **(G)** The MF Venn diagram of the common targets in the intersected targets was obtained by the four diseases and estrogen, respectively. Abbreviations: AS, atherosclerosis; BP, biological process; CC, cellular component; GO, gene ontology; MF, molecular function; NAFLD, nonalcoholic fatty liver disease; T2DM, type 2 diabetes mellitus.

As shown in Figures 2A–D, we screened ten items with *p* < 0.05 of BP, CC, and MF in each disease in GO analysis. Figures 2E–G show the Venn diagrams of the common targets involved in the overlapping targets of the four diseases and estrogen. The BP involved in the four diseases are signal transduction and negative regulation of gene expression. The CC involved in the four diseases is the plasma membrane, cytoplasm, integral component of the plasma membrane, membrane raft, and macromolecular complex. The MF involved in the four diseases are protein binding, zinc ion binding, identical protein binding, RNA polymerase II transcription factor activity, ligand-activated sequence-specific DNA binding, sequence-specific DNA binding, and steroid binding.

**FIGURE 3:**
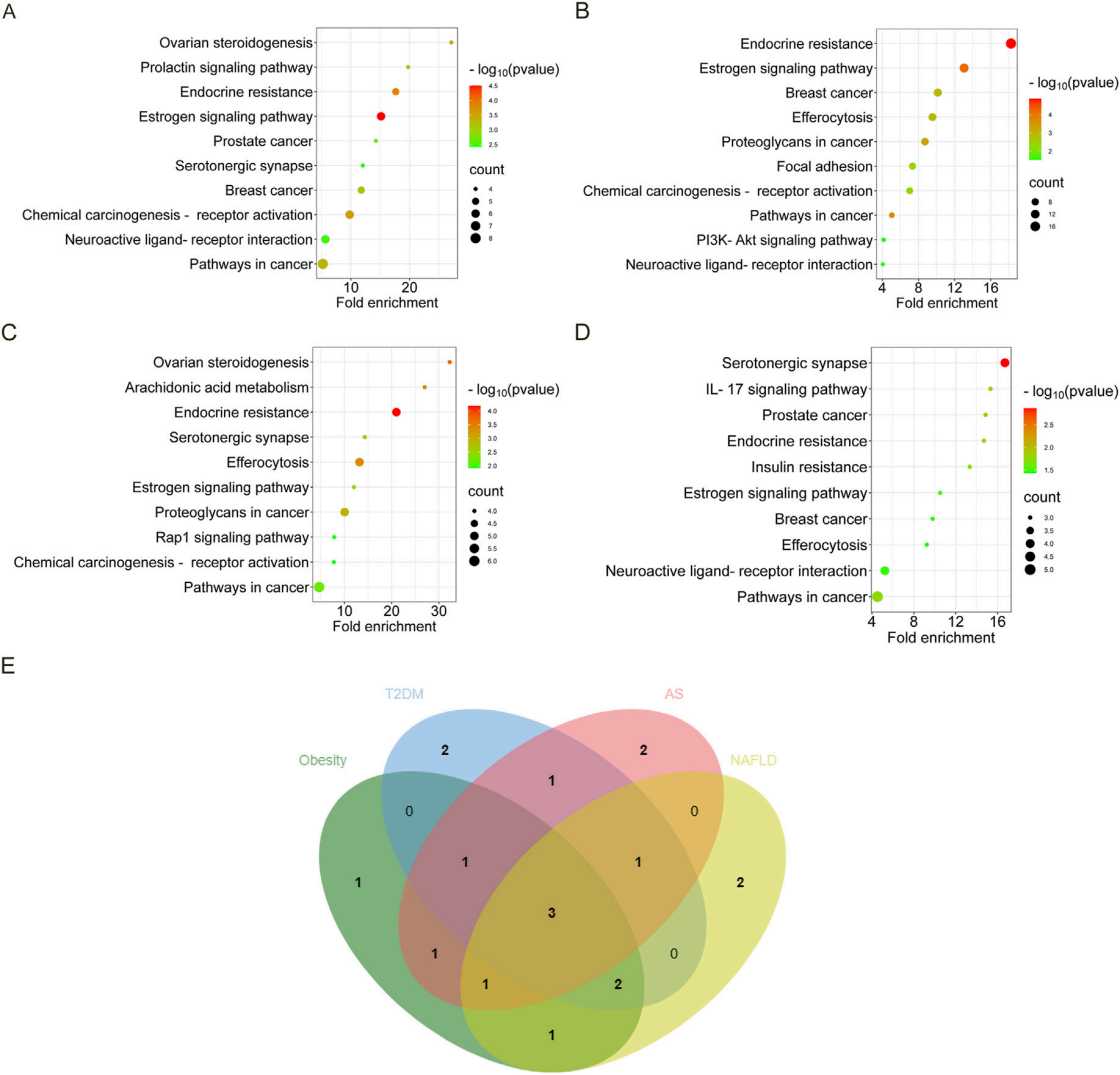
The KEGG bubble diagram and Venn diagram of the intersected targets of four diseases associated with estrogen. The abscissa is fold enrichment. **(A)** Results of the KEGG pathway analysis of the intersected targets of estrogen and obesity. **(B)** Results of the KEGG pathway analysis of the intersected targets of estrogen and T2DM. **(C)** Results of the KEGG pathway analysis of the intersected targets of estrogen and AS. **(D)** Results of the KEGG pathway analysis of the intersected targets of estrogen and NAFLD. **(E)** The KEGG Venn diagram of the intersected targets of the four diseases and estrogen were obtained, respectively. Abbreviations: AS, atherosclerosis; KEGG, Kyoto encyclopedia of genes and genomes; NAFLD, nonalcoholic fatty liver disease; T2DM, type 2 diabetes mellitus.

Regarded fold enrichment as the abscissa, the visual analysis of the KEGG pathway is presented in Figures 3A–D. A total of 17 pathways are obtained in obesity, 29 pathways in T2DM, 19 pathways in AS, and 12 pathways in NAFLD. The pathways with *p* < 0.05 involved in the four diseases are pathways in cancer, estrogen signaling pathway, and endocrine resistance. The pathways involved in the three disease processes among MIS are chemical carcinogenesis-receptor activation, neuroactive ligand-receptor interaction, efferocytosis, breast cancer, and serotonergic synapse (Figure 3E).

## 3 Validation studies of estrogen on metaflammation in MIS

### 3.1 Data selection and extraction

#### 3.1.1 Search strategy

**FIGURE 4:**
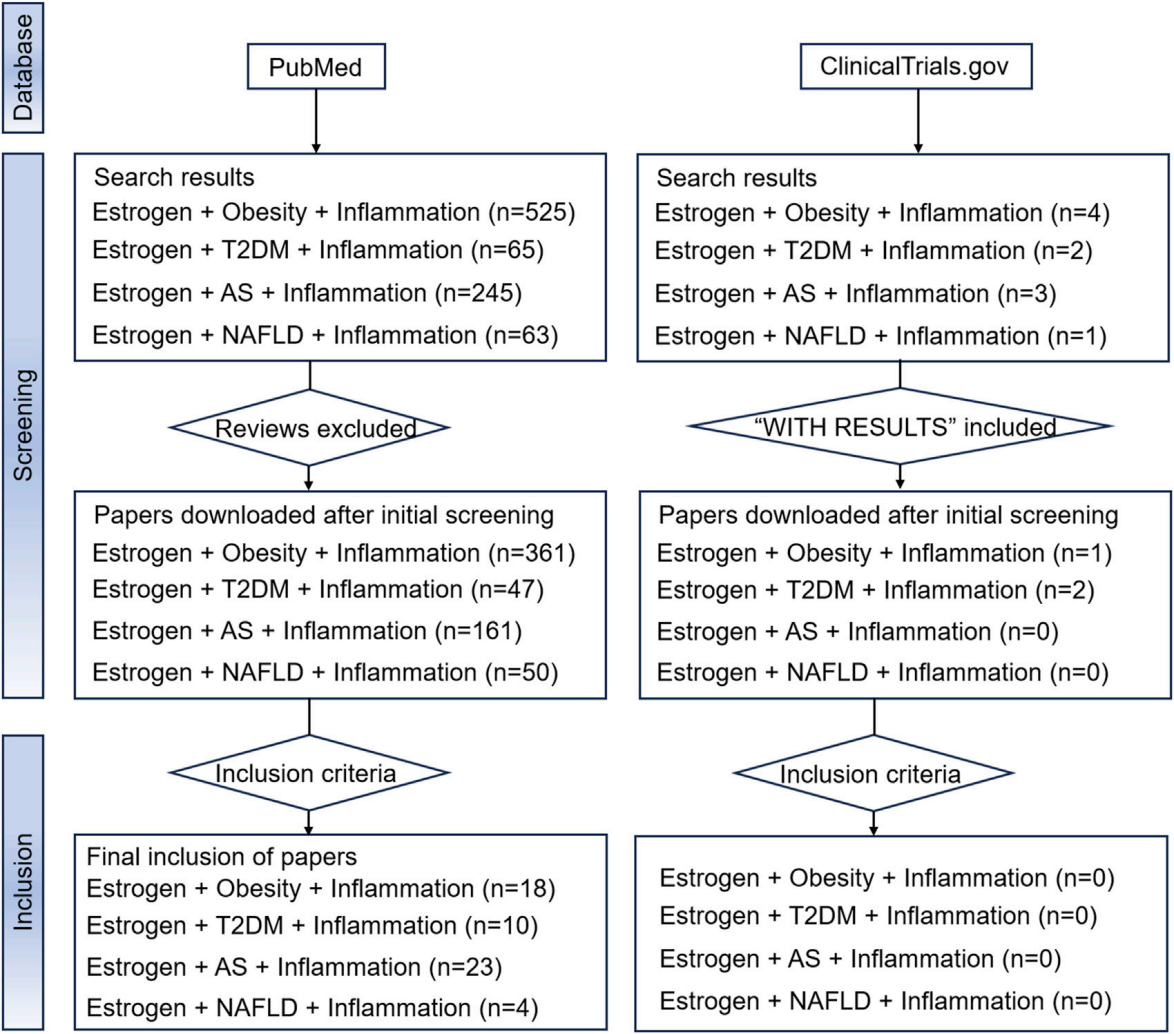
The flowchart of our search strategy. Abbreviations: AS, atherosclerosis; NAFLD, nonalcoholic fatty liver disease; T2DM, type 2 diabetes mellitus.

Due to the aim of the study, reviews, systematic reviews, and meta-analyses were excluded. Two databases, PubMed and ClinicalTrials.gov., which contain mainstream original studies and the vast majority of clinical trials, were searched. To achieve better retrieval in the PubMed database search, we used estrogen and MIS (including “obesity,” “type 2 diabetes mellitus,” “atherosclerosis,” and “non-alcoholic fatty liver disease”) as keywords, and augmented the search with the qualifier “inflammation.” Medical subject headings terms and free text words were both considered in the search. The results of the search are shown in Figure 4. The Clinicaltrials.gov search terms were Condition or disease = “Obesity/Type 2 diabetes mellitus/Atherosclerosis/Non-alcoholic fatty liver disease”, Search Term = “Inflammation,” Intervention or treatment = “Estrogen/Estradiol/Estrone/Estriol.” However, no eligible clinical trials were found.

#### 3.1.2 Eligibility criteria

We screened the retrieved studies according to the following criteria. Firstly, reviews, systematic reviews, and meta-analyses were excluded. Secondly, the application drugs were a single type of endogenous or exogenous estrogens. Other estrogens were excluded, such as phytoestrogens, environmental estrogens, and estrogens combined with progestins. Thirdly, disease models used in the studies should be relevant to obesity, T2DM, AS, and NAFLD. Furthermore, observations in these studies should include inflammation-related receptors, pathways, or inflammatory mediators. Human, animal, and cell studies were all included. Language or date restrictions were not imposed.

### 3.2 Results of validation studies

#### 3.2.1 Whether estrogen exerts anti-inflammatory or pro-inflammatory effects in obesity is controversial

Obesity is associated with white adipose tissue (WAT) inflammation, characterized by macrophages accumulated around the dead adipocytes. (Cinti et al., 2005). A high-fat diet (HFD) induced fat accumulation, increased the expression of tumor necrosis factor-alpha (TNF-α), interleukin-6 (IL-6), and monocyte chemotactic protein-1 (MCP-1), hepatocellular ballooning and the excessive infiltration of M1 macrophages in ovariectomized (OVX) female Sprague-Dawley rats (Sucedaram et al., 2021). Regulatory and senescence-related T cells were increased in HFD-fed male C57BL/6N mice than in females (Imano et al., 2023). Moreover, E2 downregulated the visceral adipose inflammation by abolishing the amount of senescence-related T cells in OVX female mice (Imano et al., 2023). HFD-fed female mice showed less immune cell infiltration in adipose tissue than weight-matched male mice fed with HFD (Nickelson et al., 2012). These studies suggested that estrogen protects against obesity-induced inflammation.

Estrogen is reported to prevent obesity-induced inflammation through various mechanisms. E2 decreased the transcription of CD68 and MCP-1 in WAT of OVX female mice fed with HFD, indicating that estrogen reduced low-grade inflammation in WAT (Shen et al., 2014). Non-OVX and OVX female mice supplemented with 17β estradiol had significantly reduced CD68 and TNF-α mRNA levels than OVX female and male mice, indicating that estrogen can protect female mice from adipose tissue inflammation (Stubbins et al., 2012). As observed in skeletal muscle-specific aromatase overexpression (SkM-Arom) female mice, E2 and E1 in skeletal muscle were significantly increased, and a physiologically relevant increased E2 concentration and reduced adipose tissue inflammation. However, the enhanced skeletal muscle estrogen level does not provide a metabolic benefit, such as alleviating IR in gonadally intact and OVX female mice with obesity (Aladhami et al., 2022). However, enhanced aromatase activity in male SkM-Arom mice obviously increased E2 in skeletal muscle, liver, adipose tissue, and circulation, which not only reduced inflammation in adipose tissue but also alleviated HFD-induced hyperglycemia, hyperinsulinemia, and glucose intolerance (Unger et al., 2023). Therefore, although E2 derived from skeletal muscle exerts anti-inflammatory effects on both female and male mice adipose tissue, there is still a difference in the impact of skeletal muscle-derived estrogen on metabolism between males and females. However, it remains unclear whether this difference exists for other sources of estrogen. Further study will assist in determining whether typical endogenous estrogen levels contribute to the observed differences.

ERα plays an essential role in the anti-inflammatory effects of estrogen in adipose tissue. Global deletion of the ERα gene (αERKO) in mice promoted the inflammation and fibrosis of adipose tissue before the onset of obesity (Davis et al., 2013). To elucidate the role of ERα in adipocytes, the adipocyte-specific knockdown of ERα (AdipoERα) mice was established. Administration of exogenous estrogen alleviated obesity and adipose tissue inflammation and fibrosis in wild-type OVX female mice, and glucose tolerance was also improved, which was not observed in OVX female AdipoERα mice (Davis et al., 2013). E2 increased the transcription of prolyl hydroxylase 3 (Phd3) via ERα, reduced the activity of hypoxia-inducible factor 1alpha (HIF-1α), and protected adipose tissue from inflammation and fibrosis (Kim et al., 2014). E2 significantly inhibited the lipopolysaccharide (LPS)-induced production of MCP-1 in adipocytes by inhibiting the p38 mitogen-activated protein kinase (p38 MAPK)/nuclear factor-κB (NF-κB) signaling cascade via ERα, thereby exerting an anti-inflammatory effect (Mu et al., 2016). Estrogen prevents weight gain, WAT inflammation in the breast, and upregulation of pro-inflammatory mediators and aromatase in the mammary gland via ERα(Bhardwaj et al., 2015). However, it remains an open question whether E2 alleviates inflammation in WAT due to weight loss or has additional anti-inflammatory mechanisms. E2 reduced obesity-induced WAT inflammation around the prostate in mice (Bhardwaj et al., 2019). E2 prevented IL-6-induced inflammation by activating both ERα and GPER and improved mitochondrial dysfunction induced by inflammation in adipocytes (Bauzá-Thorbrügge et al., 2019). The effect of estrogen on energy homeostasis was mainly mediated by ERα. However, in the case of decreased ERα expression in adipocytes, ERβ protected inflammation and fibrosis in adipose tissue (Davis et al., 2013). Therefore, we know that classical estrogen receptors (ERα and ERβ) and GPER all contribute to the anti-inflammatory effects of estrogen in adipose tissue. At the same time, they may have a primary and secondary role. The available evidence mainly focuses on ERα, and more studies on other receptors must be further carried out.

On the other hand, estrogen may aggregate inflammation induced by obesity. E2 reduced weight gain but increased circulating IL-6 levels in OVX female C57BL/6J mice fed with HFD, suggesting that E2 may not be a risk-free intervention for obesity (Mamounis et al., 2018). Additionally, when combined with LPS, E2 tended to increase the expression of inflammatory molecules such as MCP-1, IL-6, interleukin-1 beta (IL-1β), and TNF-α in adipocytes of healthy male subjects (Di Vincenzo et al., 2023). In another study of LPS as a pro-inflammatory condition, E2 increased the expression of adipose triglyceride lipase in adipocytes via ERα to alleviate obesity, which was weakened under the inflammation stimulated by LPS (Luo et al., 2017). After the treatment of estrogen, all experiment results mentioned above of the inflammatory markers appeared to be contrary to the anti-inflammatory effects of estrogen. Of course, the change of a single inflammatory cytokine cannot wholly exhibit the change of systemic inflammation. The “abnormal changes” of these inflammatory molecules may be closely related to the dose, administration route of estrogen, and the recipient.

Similarly, in human studies, estrogen did not seem to alleviate inflammation in obesity significantly. One randomized, double-blind, placebo-controlled pilot trial suggested that 12-week treatment with conjugated estrogen in obese menopausal women improved β cell function but induced no changes in systemic inflammation markers (Lovre et al., 2019). Interleukin-10 (IL-10) was significantly increased in postmenopausal women with obesity and T2DM but not in men (Subramanian et al., 2022). However, although IL-10 mRNA in WAT was significantly associated with circulating E1 rather than SHBG and E2 in premenopausal obese women, E1 did not affect IL-10 expression either in stimulated THP-1 macrophages or in primary WAT stromal vascular fraction (Subramanian et al., 2022). The elevated IL-10 levels in the WAT of obese and T2DM women relative to men were primarily attributed to obesity rather than to circulating estrogen (Subramanian et al., 2022).

It has been previously demonstrated that the benefits of estrogen on obesity, which are mediated by its regulation of metabolic pathways, may be subject to modulation by the patient’s inflammatory state. Moreover, many studies above have shown that estrogen can alleviate obesity by regulating anti-inflammatory pathways. These reports led us to consider an interesting question. What is the relationship between estrogen, inflammation, and metabolism-related signaling pathways in obesity? It may depend on the timing of estrogen administration and the inflammation status of the recipient. We speculate that estrogen is anti-inflammatory in obesity in the early stages of inflammation. With the development of inflammation in obesity, the anti-inflammatory effect of estrogen on obesity and even the protective effect of estrogen on obesity through regulating metabolism-related signaling pathways diminishes. However, the determinants of transition and the turn-off button remain unknown.

#### 3.2.2 The anti-inflammatory and cardiovascular protective effects of estrogen in T2DM are questionable

Evidence shows that estrogen plays a protective role in the inflammation status of T2DM. E2 improves the metabolic disorders and cardiovascular dysfunction caused by T2DM by improving blood lipids and blood glucose spectrum, reducing inflammatory molecules, and increasing anti-inflammatory cytokines (Azizian et al., 2018; Azizian et al., 2022). It was demonstrated that E2 induced a dose-dependent inhibition on the translocation of NF-κB in isolated apoptotic islet cells exposed to pro-inflammatory cytokines (Contreras et al., 2002). Furthermore, it was observed that E2 reversed cytochrome c release and decreased caspase-9 activity, thereby indicating that E2 inhibits apoptosis in islet cells under inflammatory conditions (Contreras et al., 2002).

However, there are still controversies on the anti-inflammatory effect of estrogen in T2DM. Estrogen deprivation did not aggregate hyperglycemia in non-obese T2DM Goto-Kakizaki (GK) rats (Apaijai et al., 2017). OVX decreased the expression of the pro-inflammatory cytokines, IL-6, TNF-α, serpin family E member 1, and C-C motif chemokine ligand 3 in female Zucker diabetic fatty rats (Martínez-Cignoni et al., 2021). Conversely, E2 supplementation abolished the downregulation of ovariectomy on these inflammatory cytokines (Martínez-Cignoni et al., 2021).

Moreover, estrogen was reported to have a pro-inflammatory effect. E2 treatment significantly increased macrophage density and plasma levels of IL-6, TNF-α, and IL-1β in OVX mice fed with HFD for 3 months (Riant et al., 2009). E2 treatment on chimeric mice grafted with bone marrow cells from ERα-deficient mice showed decreased pro-inflammatory cytokines and the same metabolic benefits, such as improved insulin sensitivity and glucose tolerance, suggesting that the benefits of E2 on metabolism were not related to the regulation of pro-inflammatory cytokines in visceral adipose tissue (Riant et al., 2009).

Much attention has been paid to the controversial cardiovascular effects of estrogen in T2DM. Estrogen depletion was observed to augment the severity of cardiac hypertrophy, precipitating cardiac inflammation and oxidative stress in female non-obese GK rats (Apaijai et al., 2017). However, additional findings are inconsistent with the cardiovascular protective effects of estrogen. Although E2 treatment partially restored the levels of TNF-α, C-reactive protein (CRP), and endothelial nitric oxide synthase (eNOS) in OVX T2DM rats to exert a specific vasoprotective effect, the effect was not as significant as that observed with a selective ERα agonist, propyl pyrazole triol (PPT) (Bansal and Chopra, 2018). Moreover, the administration of E2 was shown to reverse the beneficial effects of ovariectomy in female Zucker diabetic fatty rats, including enhanced glucose and insulin tolerance, which indicated that in the context of diabetes, estrogen was no longer capable of providing cardioprotection (Martínez-Cignoni et al., 2021).

The effects of estrogen in T2DM are anti-inflammatory or pro-inflammatory are unclear in relatively limited human studies. A meta-analysis suggested that estrogen alleviated IR and fasting blood glucose in women with T2DM, indicating a protective role for estrogen in T2DM (Salpeter et al., 2006). However, estrogen level seems to be a risk factor for T2DM in men. As demonstrated by a nested case-control study, sex hormones or the combinations of SHBG and sex hormones in female subjects had no risk correlation with T2DM (Hu J. et al., 2016). However, males with low SHBG and high estradiol levels showed a significantly enhanced risk of T2DM (Hu J. et al., 2016).

However, most studies are sole observations of changes in inflammatory cytokines. There is an urgent need to elucidate estrogen’s targets and molecular mechanisms in inflammation in T2DM.

#### 3.2.3 There is strong evidence that estrogen has an anti-inflammatory effect on AS

Estrogen exerts anti-inflammatory effects directly or indirectly in AS. Both endogenous and pharmacological estrogen might indirectly regulate the inflammatory state of AS by decreasing blood lipids such as total cholesterol, triglyceride, and low-density lipoprotein cholesterol (Meng et al., 2021; Folahan et al., 2023; Meng et al., 2023). E2 inhibited the induction of circulating E-selectin, intercellular adhesion molecule-1 (ICAM-1), and vascular cell adhesion molecule-1 (VCAM-1) in human umbilical vein endothelial cells in postmenopausal women with coronary artery disease, which was associated with decreased cellular adhesion molecules levels (Caulin-Glaser et al., 1996; Caulin-Glaser et al., 1998). And pretreatment with E2 did not significantly impact the TNF-α-induced upregulation of pro-inflammatory molecules, including ICAM-1 and VCAM-1 in the human umbilical vein endothelial cells (HUVECs) (Chakrabarti and Davidge, 2012; 2013). It was reported that E2 inhibited leukocyte adhesion to endothelial cells by inhibiting the secretion of pro-inflammatory molecules, interleukin-8 (IL-8), and MCP-1 (Rodríguez et al., 2002). Estradiol valerate significantly attenuated oxidized lipid-induced vascular inflammation and atherosclerosis by alleviating oxidative stress and inhibiting the TNF-α signaling (Folahan et al., 2023). MMP-9, a pro-inflammatory mediator produced by vascular smooth muscle cells or macrophages, was decreased by estradiol in OVX mice fed with HFD and female cynomolgus macaques consuming atherogenic diets (Sophonsritsuk et al., 2013; Meng et al., 2023). E2 enhanced the production of the anti-inflammatory cytokine nitric oxide (NO) in endothelial cells by downregulating the endothelial nitric oxide synthase (eNOS). E2 restored OVX-induced decreased eNOS, increased inducible nitric oxide synthase (iNOS) protein expression, and decreased IL-6 and TNF-α in OVX rats (Lamas et al., 2015). CRP is an acute phase reactant during inflammation, and its level will produce corresponding changes in AS. Pretreatment with E2 decreased the expression of IL-8, IL-6, ICAM-1, and VCAM-1 induced by recombinant human CRP (rhCRP) in human aortic endothelial cells (Cossette et al., 2013).

The anti-inflammatory effects of estrogen in various cells in AS have been demonstrated to be mainly achieved via ERα, and the anti-inflammation effects of estrogen mediated by ERβ and GPER have been recognized recently. It was reported that E2 did not influence the nuclear translocation of NF-κB in TNF-α-treated human coronary SMCs but dose-dependently inhibited the transcription of ICAM and attenuated P65-dependent activation of ICAM-1-CAT constructs via ERα (Speir et al., 2000). In the presence of stressors such as cytomegalovirus and TNF-α, activated NF-κB may repress E2-and ER-dependent transcription by binding to p300 (Speir et al., 2000). E2 reduced the production of LPS-stimulated MCP-1 in vascular smooth muscle cells (VSMCs) by inhibiting the p38 MAPK/NF-κB signaling cascade via ERα, thereby decreasing LPS-induced cell migration and exerting an anti-inflammatory effect (Jiang et al., 2010). The suppressor of cytokine signaling (SOCS) has anti-inflammatory and metabolic regulation effects in many diseases, including AS (Carow and Rottenberg, 2014; Pedroso et al., 2019). In OVX ApoE null mice and RAW264.7 cells, E2 enhanced the expression of suppressor of cytokine signaling 3 (SOCS3) in atherosclerotic plaques via ERα, thereby inhibiting the activation of the Janus kinase/signal transducers and activators of transcription (JAK/STAT) pathway, reversing the downregulation of ATP-binding cassette transporter A1 (ABCA1) and increasing cholesterol efflux in macrophages, to attenuate the formation of foam cells (Liang et al., 2013). Estrogen significantly reduced the serum levels of IL-6, IL-1β, MCP-1, and TNF-α in OVX LDLR^−/−^ mice fed with HFD, possibly through inhibiting the toll-like receptor 4 (TLR4) signaling via ERα(Meng et al., 2023). As demonstrated in HUVECs and OVX ApoE^−/−^ mice, estrogen upregulated ERα and then induced autophagy to inhibit the NLR family pyrin domain containing 3 (NLRP3) inflammasome, which indirectly suppressed the secretion of IL-1β and IL-18 and reduced inflammation and pyroptosis (Meng et al., 2021). Additionally, E2 may downregulate sirtuin 1 (SIRT1) levels in rat VSMCs and human peripheral blood monocytes via ERα, which may be due to the activation of AMP-activated protein kinase (AMPK) (Toniolo et al., 2013). E2 increased protein S-nitrosylation mediated by eNOS rather than iNOS or neuronal nitric oxide synthase (nNOS) via ERα in HUVECs, which prevented the upregulation of intercellular adhesion molecule-1 induced by angiotensin II. Exogenous E2 was demonstrated to increase endothelial protein S-nitrosylation in female Sprague-Dawley rats (Chakrabarti et al., 2010).

It remains unclear whether ERβ is an essential receptor in the anti-inflammatory effect of estrogen in AS. However, it has been reported that ERβ-related proteins changed in AS. NME/NM23 nucleoside diphosphate kinase 2 (NM23-H2), a metastasis suppressor, was considered an ERβ-associated protein, and its expression and nuclear localization were increased in estrogen-treated VSMCs (Rayner et al., 2007). Heat shock protein 27 (HSP27) was also identified as ERβ-associated. E2 caused a dose-dependent release of HSP27 into the medium of macrophages, and extracellular HSP27 competitively inhibited the acetylated low-density lipoprotein (acLDL) binding to scavenger receptor-A to reduce the formation of foam cells and inflammation (Rayner et al., 2008).

The role of GPER in the anti-inflammatory effect of estrogen in AS has received more and more attention. GPER is predominantly located in the nucleus of human endothelial cells (Chakrabarti and Davidge, 2012). Activating GPER attenuated TNF-α-induced inflammation, characterized by the expression of ICAM-1 and VCAM-1, while it may not be related to the activation of NF-κB (Chakrabarti and Davidge, 2012). Interestingly, estradiol lacked the anti-inflammatory activities of GPER agonists, and the simultaneous activation of the classical ERs blocked the anti-inflammatory effects of the selective GPER agonist G-1 (Chakrabarti and Davidge, 2012). In human endothelial cells of an hTERT-immortalized umbilical vein endothelial (TIVE) cell line expressing GPER, estrogen inhibited the production of TNF-α-stimulated thromboxane A2 (Meyer et al., 2015). OVX increased prostanoid-mediated contraction in wild-type female mice (Meyer et al., 2015). In contrast, this phenomenon was not observed in GPER knockout mice, suggesting endogenous estrogen inhibited vasoconstrictor prostanoid activity via GPER in diet-induced vascular inflammation (Meyer et al., 2015). Available limited evidence suggests that estrogen can benefit inflammation via both classical ERs and GPER, which is good news. However, their effects are parallel, synergistic, or antagonistic and remain to be elucidated.

Other anti-inflammatory signaling pathways of estrogen in AS have been extensively investigated. However, whether ERs or GPERs are involved remains to be elucidated. In human endothelial cells, pretreatment with E2 did not significantly impact TNF-induced upregulation of pro-inflammatory molecules, ICAM-1 and VCAM-1, because the pro-inflammatory effects of TNF were mediated by TNF receptor 1 (TNFR1). In contrast, E2 pretreatment increased TNF receptor 2 (TNFR2) levels in these cells (Chakrabarti and Davidge, 2013).

Novel ideas about the effects of estrogen on AS should be considered. The current study provided evidence for the time hypothesis of estrogen therapy (ET) in the cynomolgus monkey model. ET initiated soon after menopause inhibited the accumulation of macrophages in the carotid artery, but it was not observed when estradiol was given after several years of estrogen deficiency (Sophonsritsuk et al., 2013). Therefore, the anti-inflammatory effect of estrogen on AS may be related to the timing of application. The optimal timing of ET for AS remains to be established.

Further clinical trials are required to deepen our understanding of estrogen’s anti-inflammatory role in AS. An observational study showed that postmenopausal women exhibited higher expression of membrane-bound TNF-α on CD14 monocytes and adhesion molecules than menopausal women (Figueroa-Vega et al., 2015). Since estradiol and free estradiol levels positively correlate with CRP and fibrinogen, estradiol seems pro-inflammatory in older men with coronary artery disease (Barud et al., 2010). However, a randomized, double-blind, placebo-controlled clinical trial on postmenopausal women demonstrated that soluble ICAM-1 and homocysteine levels were inversely correlated with estrogen, indicating that estrogen had anti-inflammatory effects on postmenopausal women. In contrast, CRP was positively associated with estrogen, which may be explained by the first-pass effect (Karim et al., 2010). Therefore, whether CRP is the contributor or consequence of AS remains elucidated.

#### 3.2.4 Current evidence for the anti-inflammatory effect of estrogen in NAFLD is insufficient

Some evidence supports that estrogen has an anti-inflammatory effect on NAFLD. OVX ApoE^−/−^ mice were prone to nonalcoholic steatohepatitis (NASH) induced by a Western diet. At the same time, estradiol offers protection against NASH by reducing the phosphorylation of c-Jun N-terminal kinase (JNK) to inhibit NF-κB (Araujo et al., 2023). Estrogen-related receptor α (ERRα) is an orphan nuclear receptor homologous to ERα and regulates metabolism (Tripathi et al., 2020). It was found that the tamoxifen-suppressed hepatic very low-density lipoproteins-triglyceride (VLDL-TG) secretion, as well as hepatic apolipoprotein B (ApoB), microsomal triglyceride transfer protein (Mttp), and phospholipase A2 G12B (Pla2g12b) expression in female C57BL/6 mice were rescued by enforced ERRα expression, implying that decreased ERRα expression was a contributing factor to tamoxifen-induced NAFLD (Yang M. et al., 2020). Treatment of primary hepatocytes with E2 enhanced the expression of ERRα and VLDL-related genes in female flox mice, such as ApoB, Mttp, and Pla2g12b. At the same time, the induction was blocked in primary hepatocytes from liver-specific ERRα-deficient (ERRαLKO) mice, indicating that ERRα-mediated hepatic VLDL-TG secretion protected against NAFLD induced by estrogen deficiency in female mice (Yang M. et al., 2020). In a word, ERRα was an indispensable mediator of estrogen/ERα signaling and regulated hepatic VLDL secretion through the coordinated control of the target genes Mttp, ApoB, and Pla2g12b (Yang M. et al., 2020). Estrogen exerted an anti-inflammatory effect in the liver directly via regulating the expression of formyl peptide receptor 2 (FPR2) (Lee et al., 2022). FPR2, a downstream target of estrogen, had a binding site for ER, which was more likely to be ERα and alleviated lipid accumulation in hepatocytes by influencing phosphatidylethanolamine N-methyltransferase (PEMT) and ameliorates liver injury (Lee et al., 2022). These studies mostly showed that estrogen alleviates inflammation in the liver mainly by regulating lipid metabolism pathways in hepatocytes. Nevertheless, the impact of estrogen on hepatic immune cells, such as Kupffer cells associated with NAFLD/NASH, remains largely unstudied. Furthermore, the function of ERs related to NAFLD remains unclear.

## 4 Gaps between *in silico* study and validation studies

Since any disease in MIS is not entirely equivalent to metaflammation, to make the results closer to the effect of estrogen on metaflammation, we mainly discuss the common targets of estrogen for obesity, T2DM, AS, and NAFLD predicted by network pharmacology. Current validated studies are summarized in Table 1.

**TABLE 1:** The validated mechanisms of estrogen on metaflammation in MIS.

Diseases	Model	Treatment	Mechanism	Inflammation	Reference
Obesity	Female SkM-Arom C57BL/6 mice fed with HFD	-	E1, E2 (↑) CD68, CD11c, CD206, TLR2 (↓)	↓	Aladhami et al. (2022)
Obesity	Male SkM-Arom C57BL/6 mice fed with HFD	-	E2 (↑) TNF-α, MCP-1, CD68, CD11c, CD206 (↓)	↓	Unger et al. (2023)
Obesity	Adipocytes from female Sprague-Dawley rats induced with LPS	E2 (10^–9^, 10^–8^, 10^–7^, 10^–6^ M) for 24 h	MCP-1 (↓)	↓	Mu et al. (2016)
Obesity	Mammary glands from OVX C57BL/6J female mice fed with HFD	Subcutaneous implantation of E2 pellet (0.1 mg) for 60-day continuous release	F4/80, MCP-1, COX2, PGE2, TNF-α, IL-1β, aromatase (↓)	↓	Bhardwaj et al. (2015)
Obesity	Periprostatic white Adipose tissue from male C57BL/6J mice fed with HFD	Subcutaneous implantation of E2 pellet (0.25 mg) for 60-day continuous release	CD68, MCP-1, TNF, TGFβ1, IFNG, IL-6, IL-1β (↓)	↓	Bhardwaj et al. (2019)
T2DM	Islets cultured for 4 days in the presence of a combination of recombinant human IFN-γ, IL-1β and TNF-α	E2 (10^–7^, 10^–6^, 10^–5^, 10^–4^ M)	Cytochrome c, caspase 9 (↓)	↓	Contreras et al. (2002)
T2DM	Four-week-old C57BL6/J female mice fed with HFD for 12 weeks	E2 (80 μg/kg) for 60 days	IL-6, TNF-α, IL-1β (↑)	↑	Riant et al. (2009)
T2DM	OVX-T2DM female Sprague Dawley rats	E2 (10 μg/kg) for 7 weeks	TBARS, TNF-α, CRP (↓) eNOS, SOD (↑)	↓	Bansal and Chopra (2018)
AS	OVX LDLR^−/−^ mice fed with HFD	Estradiol (0.13 mg/kg) for 90 days	IL-1β, IL-6, TNF-α, MCP-1, MMP-9, TLR4, NF-κB p65 (↓) ERα (↑)	↓	Meng et al. (2023)
AS	RAW264.7 cells induced with LPS	Estradiol (10 nM) for 24 h	TLR4, MYD88, TRAM, IRF3, IL-1β, MCP-1, MMP-9 (↓) TIMP1 (↑)	↓	Meng et al. (2023)
AS	OVX healthy female Wistar rats fed with TSO or TPO	Estradiol valerate (0.2 mg/kg) for 12 weeks	TNF-α, MDA (↓) CAT, SOD, GSH (↑)	↓	Folahan et al. (2023)
AS	HUVECs treated with TNF	E2 (10 nM) pre-treated for 24 h	TNFR2 (↑)	-	Chakrabarti and Davidge (2013)
AS	Female cynomolgus macaques consuming atherogenic diets (initiated at 1 month, early menopause)	Oral micronized estradiol (human equivalent dose of 1 mg/d) for 8 months	IFN-γ, IL-4, MCP-1, TNF-α, IL-10, MMP-9 (↓)	↓	Sophonsritsuk et al. (2013)
AS	Female cynomolgus macaques consuming atherogenic diets (initiated at 54 months, late menopause)	Oral micronized estradiol (human equivalent dose of 1 mg/d) for 8 months	MCP-1, TNF-α, IL-10, MMP-9 (↓)	-	Sophonsritsuk et al. (2013)
AS	OVX female Wistar rats	E2 (0.5 μg/kg) for 14 days	iNOS, IL-6, TNF-α (↓) eNOS(↑)	↓	Lamas et al. (2015)
AS	HAEC treated with rhCRP	E2 (10^–8^ and 10^–9^ M)	CRP, IL-6, IL-8, VCAM-1, ICAM-1 (↓)	↓	Cossette et al. (2013)
AS	VSMCs from female Sprague-Dawley rats	E2 (10^–9^, 10^–8^, 10^–7^, 10^–6^ M) for 24 h	MCP-1 (↓)	↓	Jiang et al. (2010)
AS	RAW264.7 cells treated with IFN-γ	E2 (100 nM) for 12 h	SOCS3, ABCA1 (↑)	↓	Liang et al. (2013)
AS	VSMCs from normoglycemic rats	E2 (10 nM) for 0, 6, 24 h	SIRT1 (↓)	-	Toniolo et al. (2013)
AS	HUVECs treated with AngⅡ	E2 (1 and 10 nM) for 24 h	ICAM-1 (↓)	↓	Chakrabarti et al. (2010)
AS	HCASMCs	Estradiol (100 nM) for 24 h	NM23-H2 (↑)	-	Rayner et al. (2007)
AS	U937 cells	E2 (0.1, 10, 100 nM) for 24 h	HSP27 (↑)	-	Rayner et al. (2008)
AS	TIVE cells treated with TNF	E2 (100 nM) for 24 h	Thromboxane A2 (↓)	-	Meyer et al. (2015)
AS	A total of 222 postmenopausal women (107 in estradiol group)	Oral unopposed micronized E2 (1 mg/d) for 2 years	sICAM-1, homocysteine (↓) CRP (↑)	↓	Karim et al. (2010)
NAFLD	Female Flox and ERRαLKO hepatocytes	E2 (100 nM) for 36 h	ApoB, Mttp, Pla2g12b (↓)	↓	Yang et al. (2020b)
NAFLD	Male C57BL/6 mice fed with CDAHFD for 12 weeks	E2 pellet (0.36 mg) for 13 weeks	FPR2 (↑) α-SMA, Col1α1, TNF-α, IL-6, F4/80 (↓)	↓	Lee et al. (2022)
NAFLD	OVX female C57BL/6 mice fed with CDAHFD for 12 weeks	E2 pellet (0.36 mg) for 13 weeks	FPR2 (↑)	↓	Lee et al. (2022)

Abbreviations: AS, atherosclerosis; ABCA1, ATP-binding cassette transporter A1; AngⅡ, angiotensin Ⅱ; ApoB, apopolipoprotein B; CAT, catalase; Col1α1, collagen 1 alpha 1; COX2, cyclooxygenase-2; CRP, C-reactive protein; E1, estrone; E2, 17β-estradiol; eNOS, endothelial nitric oxide synthase; ERRαLKO, liver-specific Estrogen-related receptor alpha -deficient; ERα, estrogen receptor alpha; FPR2, formyl peptide receptor 2; GSH, glutathione; HAEC, human aortic endothelial cell; HCASMCs, human coronary artery smooth muscle cells; HFD, high-fat diet; HSP27, heat shock protein 27; HUVECs, human umbilical vein endothelial cells; ICAM-1, intercellular adhesion molecule-1; IFNG, interferon gamma; IFN-γ, interferon-γ; IL-10, interleukin-10; IL-1β, interleukin-1, beta; IL-6, interleukin-6; IL-8, interleukin-8; iNOS, inducible nitric oxide synthase; IRF3, interferon regulatory factor 3; LPS, lipopolysaccharide; MCP-1, monocyte chemotactic protein-1; MDA, malondialdehyde; MMP-9, matrix metalloproteinases-9; Mttp, microsomal triglyceride transfer protein; MYD88, myeloid differentiation primary response protein 88; NAFLD, nonalcoholic fatty liver disease; NF-κB p65, nuclear factor-κB p65; NM23-H2, NME/NM23 nucleoside diphosphate kinase 2; OVX, ovariectomized; PGE2, prostaglandin E2; Pla2g12b, phospholipase A2 G12B; rhCRP, recombinant human C-reactive protein; sICAM-1, soluble intercellular adhesion molecule-1; SIRT1, sirtuin 1; SkM-Arom, skeletal muscle-specific aromatase overexpression; SOCS3, suppressor of cytokine signaling 3; SOD, superoxide dismutase; T2DM, type 2 diabetes mellitus; TBARS, thiobarbituric acid reactive species; TGFβ1, transforming growth factor beta 1; TIMP1, tissue inhibitor of metalloproteinase 1; TIVE, hTERT-immortalized umbilical vein endothelial; TLR2, toll-like receptors; TLR4, toll-like receptor 4; TNF, tumor necrosis factor; TNFR2, tumor necrosis factor receptor 2; TNF-α, tumor necrosis factor-alpha; TPO, thermoxidized palm oil; TSO, thermoxidized soya oil; VCAM-1, vascular cell adhesion molecule-1; VSMCs, vascular smooth muscle cells; α-SMA, alpha-smooth muscle actin.

**FIGURE 5:**
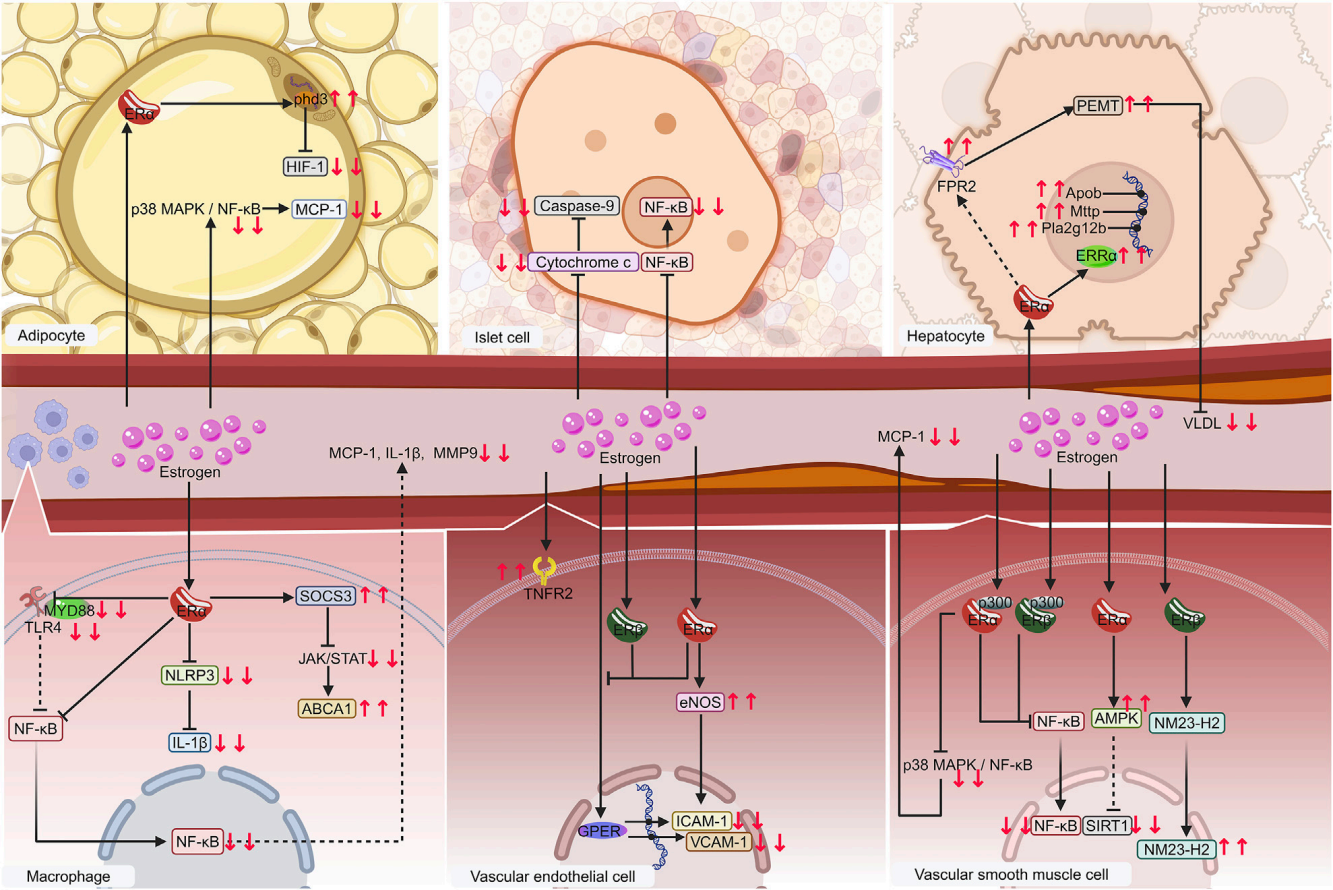
Diagram of validated mechanisms of estrogen on metaflammation in MIS. Macrophages, vascular endothelial cells, vascular smooth muscle cells, especially adipocytes, islet cells, and hepatocytes, are significant contributors to MIS. Estrogen regulates metaflammation mainly by affecting the NF-κB pathway via binding to receptors such as ERα, ERβ, ERRα, and GPER on these cells. The following proteins, such as cytochrome c, FPR2, and TNFR2, regulate IL-1β, MCP-1, MMP-9, VLDL, ICAM-1, and VCAM-1, etc. are regulated in this process. Solid lines denote items that have already been verified. The dashed lines indicate possible mechanisms. Downward red arrows indicate reduction or suppression, while upward red arrows indicate an increase or promotion. Gradient arrows indicate nuclear translocation. Abbreviations: ERRα, estrogen-related receptor alpha; ERα, estrogen receptor alpha; ERβ, estrogen receptor beta; FPR2, formyl peptide receptor 2; GPER, G protein-coupled estrogen receptor; ICAM-1, intercellular adhesion molecule 1; IL-1β, interleukin-1 beta; MCP-1, monocyte chemoattractant protein-1; MIS, metabolic inflammatory syndrome; MMP-9, matrix metalloproteinases-9; NF-κB, nuclear factor kappa-B; TNFR2, tumor necrosis factor receptor 2; VCAM-1, vascular cell adhesion molecule 1; VLDL, very low-density lipoproteins.

The importance and mechanisms of ERα has been validated (Figure 5). Estrogen exerts its effect on metabolic inflammation in VSMCs, endothelial cells, macrophages, adipocytes, and hepatocytes via ERα. ERα is likely the primary receptor of estrogen in islet cells, which is consistent with the prediction of ERα as a common target for the four diseases through network pharmacology.

The ERβ predicted by network pharmacology is a common estrogen target in obesity, T2DM, and AS, consistent with existing studies. E2 could increase energy expenditure and thus reduce adiposity through ERβ signaling (Bjune et al., 2022). Furthermore, ERβ may regulate glucose homeostasis, fibrosis, and inflammation in female AdipoERα mice (Bjune et al., 2022). The activation of ERβ contributes to some extent to vascular protection (Novella et al., 2019). However, most mechanistic studies are focused on ERα, and the molecular mechanisms of ERβ in metabolic inflammation are far from being fully understood. Additionally, clinical trials on novel drugs targeting ERβ are lacking. Consequently, studies are urgently needed to elucidate the mechanisms of ERβ in metabolic inflammation and the pharmacological potential of its selective ligands.

GPER plays a vital role in regulating metabolic homeostasis in males and females. Many of estrogen’s beneficial effects on the non-reproductive system are related to anti-inflammatory effects, some of which are mediated by GPER (Arterburn and Prossnitz, 2023). Blocking the classical ERs did not wholly block the protective effect of estrogen, indicating that GPER may be involved (Ullrich et al., 2008; Xu et al., 2023). Activation of classical ERs hampers the anti-inflammatory effect of the selective GPER agonist G-1 in endothelial cells, suggesting that the effects of GPER and classical ERs may be opposite in some circumstances (Chakrabarti and Davidge, 2012). The beneficial effects of GPER in obesity and diabetes make it a promising therapeutic target through enhancing mitochondrial biogenesis and reducing the expression of genes involved in inflammation, which receive increasing attention (Prossnitz and Barton, 2023). The development of ligands modulating the activity of the GPER receptor, including E2, selective estrogen receptor modulators (SERMs), phytoestrogens, bisphenol A, G1, and other compounds, provide novel strategies for metabolic diseases (Żabińska et al., 2024).

Moreover, ERRα contributes to the secretion of liver VLDL downstream of ERα signaling (Yang M. et al., 2020). GPER and ERRα are involved in the anti-inflammation effects of estrogen but have not been predicted by network pharmacology, suggesting that the target prediction of network pharmacology has certain limitations.

MMP-9 is predicted to play a significant role in the influence of estrogen on MIS. Recent studies suggest that matrix metalloproteinases (MMPs) are linked to the physiological and pathological aspects of obesity or metabolic syndrome. MMP-9, a member of the MMP family, has been proposed as a predictor of endothelial dysfunction (Zhang, 2022). While MMP-9 is only verified in AS as a downstream marker of estrogen in AS, its regulation by estrogen in obesity, T2DM, and NAFLD demands further investigation.

In addition to these experimentally validated targets, network pharmacology offers promising targets or pathways that are important for the effects of estrogen on metaflammation in MIS. Combined with the importance ranking of degree value determination in network pharmacology and the above studies, we believe that the following molecules may be the therapeutic targets of estrogen on metaflammation in MIS.

PTGS2, encoding cyclooxygenase 2 (COX2), is an essential molecule in ferroptosis (Zhou et al., 2021). The marker of ferroptosis is lipid peroxidation (Jiang et al., 2021), and dyslipidemia is one of the initial factors of metaflammation. Moreover, ferroptosis has been reported to be closely related to obesity (He et al., 2023), T2DM (Miao et al., 2023), AS (Wang et al., 2022), and NAFLD (Tsurusaki et al., 2019). However, little studies have linked estrogen, ferroptosis, and metaflammation. We speculate that estrogen can inhibit ferroptosis in MIS through unknown mechanisms, indirectly affecting metaflammation.

The IGF1R signaling regulates many pathways related to metabolism and inflammation (Pérez-Matute et al., 2022). IGF-1R inhibitors are now being investigated to treat obesity-related endocrine cancers (Zhong et al., 2023). However, the influence of estrogen on IGF1R signaling remains unclear and warrants further investigation.

SHBG, as a high-affinity binding protein of estrogen, can affect the concentration of free estrogen and then regulate the concentration and biological effects of estrogen in the target organ (Mendel, 1989). The SHBG level is reported to be related to T2DM, obesity, and liver diseases (Narinx et al., 2022), which may be why network pharmacology methods predict it as a target for estrogen in MIS. However, the effects of estrogen-based on SHBG are mainly investigated in reproductive system diseases. Based on the above evidence, the interaction between SHBG and estrogen plays a role in metaflammation in MIS.

The gene CNR1 encodes cannabinoid receptor 1 (CB1R), and CNR2 encodes cannabinoid receptor 2 (CB2R). They are expressed in the cardiovascular system, adipose tissue, gastrointestinal tract, pancreatic β-cell, etc. (Liu et al., 2021). CNR1 and CNR2 are essential in the endocannabinoid system (ENS) and regulate metabolic disorders and energy homeostasis (Aguilera Vasquez and Nielsen, 2022). Therefore, estrogen may be able to target ENS, thereby inhibiting the initiating factors of metaflammation by regulating dietary behavior. However, whether the regulation of inflammation is the downstream effect of ENS remains to be investigated.

## 5 The impact of estrogen on inflammation in other metabolic disorders

Inflammation also occurs in many other metabolic diseases besides MIS, such as hemochromatosis, mitochondrial diseases, Wilson disease, phenylketonuria, and lysosomal storage disorders. Furthermore, estrogen plays a role in inflammation in these diseases through various mechanisms.

Here are some inspiring evidence. Estrogen was reported to inhibit the expression of hepcidin, a central regulator of iron homeostasis, through anti-inflammatory pathways, thereby reducing the incidence of hemochromatosis (Yang L. et al., 2020; Nemeth and Ganz, 2023). Duchenne muscular dystrophy is induced by mutations in the dystrophin gene and exhibits mitochondrial dysfunction, as well as overproduction of reactive oxygen species. Estrogen alleviated functional and metabolic perturbations in female mice with Duchenne muscular dystrophy through upregulating proteins involved in mitochondrial dynamics and metabolism (Timpani et al., 2024). Decreased expression of ESR1 in muscles from men, women, and animals is associated with metabolic dysfunction through regulating mitochondrial form and function (Hevener et al., 2021). Multiple studies indicated that estrogen may benefit cellular diseases secondary to mitochondrial involvement, such as neurodegenerative, cardiovascular, inflammatory, and metabolic syndromes (Suliman and Piantadosi, 2016). Estradiol significantly increased copper uptake at the cellular level in healthy humans, suggesting that it could help maintain copper availability to meet metabolic demands (Arredondo et al., 2010). Although there is little report on the effects and mechanisms of estrogen in Wilson disease, sex differences in the clinical phenotypes of Wilson disease still suggest that estrogen plays an essential role in Wilson disease (Ferenci et al., 2019; Dev et al., 2022). Female patients with phenylketonuria have a higher prevalence of overweight and obesity than males, suggesting that estrogen may contribute to the difference (Tankeu et al., 2023). It has also been reported that there is no evidence of systemic low-grade inflammation in adult patients with early-treated phenylketonuria, suggesting that estrogen may regulate phenylketonuria through mechanisms other than affecting inflammation (Giret et al., 2023). Lysosomal storage disease is caused by defects in proteins associated with lysosomal function, which has more than 70 types, such as Fabry disease, Gaucher disease, Pompe disease, and Niemann-Pick disease type C (NPC) (Keyzor et al., 2023). Male patients with Fabry disease typically present with more severe clinical manifestations, such as chronic kidney disease, than females, suggesting the crucial role of estrogen (Coelho-Ribeiro et al., 2024). NPC, clinically presented as a progressive neurodegenerative disorder, is usually caused by mutations in the lysosomal integral membrane protein NPC1. Estradiol significantly delayed the onset of neurological symptoms in both male and female *Npc1* ^−/−^ mice, increased Purkinje cell survival, and prolonged the lifespan (Chen et al., 2007).

Estrogen’s role in inflammation in these metabolic diseases has aroused increasing interest in recent years, which will provide new clues for elucidating estrogen’s mechanisms of metaflammation.

## 6 Future perspectives

The targets of estrogen on metaflammation need to be precisely identified. We predicted possible targets of estrogen in MIS by network pharmacology, while these results are derived from currently available studies. The upregulation or downregulation effects of estrogen on these targets and their mechanisms need to be further investigated. Their potential as pharmacological targets for MIS needs to be validated, especially.

Metaflammation is a pathological status shared by many diseases. Furthermore, estrogen has been demonstrated to alleviate inflammation in multiple disorders through various pathways besides the four diseases in MIS. Hence, we need to elucidate the common inflammatory mechanism of diseases related to metaflammation, including MIS, and their possible estrogenic targets.

Whether estrogen has anti-inflammatory or pro-inflammatory effects on metaflammation in MIS should be confirmed. At present, most studies suggest that estrogen has an anti-inflammatory effect in MIS, which means estrogen is assumed to possess positive effects on metaflammation. However, as shown in our results, the effects of estrogen on the inflammation of MIS are more studied in AS, but less in the other three diseases, and most of them focus on the changes of inflammatory molecules. Additionally, since researchers pay less attention to estrogen as a therapeutic drug for MIS, there are few observational studies and clinical trials. Primarily, high-quality clinical trials with large sample sizes are lacking. Therefore, more studies on T2DM, NAFLD, and obesity, as well as further studies about the specific mechanisms and clinical trials with large sample sizes of pro- or anti-inflammatory effects of estrogen in MIS, are urgently needed. High-quality clinical trials with large sample sizes also contribute to distinguishing whether estrogen has pro- or anti-inflammatory effects due to doses, administration routes, patients, or timing at different stages in disease progression.

In addition to the above future research directions, the following interesting questions are also worthy of exploration. Since the onset and progression of metaflammation are associated with the immune system, efforts should be made to determine the effects of estrogen on immune cells, such as Kuffer cells, macrophages, and neutrophils in metaflammation. Gender differences exist in the development and severity of metaflammation. What is the reason for gender difference in metaflammation? Do phytoestrogens have therapeutic effects on metaflammation? Does estrogen have the same effect in male patients? Studies have shown that hormone imbalance is associated with metaflammation, and the focus should be on restoring the balance of hormones rather than just the change of estrogen, which leads to inflammation. Therefore, what is the effect and mechanism of estrogen to testosterone ratio on metaflammation? Will the proportional relationship between estrogen and other sex hormones affect metaflammation? Answers to these questions will advance precision medicine, enhance quality of life, and provide novel therapeutic strategies and pharmacological targets for MIS.
